# Addressing Peritoneal Dialysis: In Vitro PD Models, In Vivo Rodent PD Model, Clinical Biobanks, and Underutilization of PD

**DOI:** 10.1155/2016/4964316

**Published:** 2016-01-03

**Authors:** Robert H. J. Beelen, Donald J. Fraser, Peter Rutherford, Janusz Witowski

**Affiliations:** ^1^Department of Molecular Cell Biology and Immunology, VU University Medical Center, 1007 MB Amsterdam, Netherlands; ^2^Cardiff University School of Medicine, Cardiff, UK; ^3^Baxter World Trade, Lessines, Belgium, presently Quintiles, Reading, UK; ^4^Department of Pathophysiology, Poznan University of Medical Science, Poznan, Poland

Renal replacement therapy (RRT) is necessary for the survival of patients with end-stage renal disease (ESRD) both prior to kidney transplantation or in patients where kidney transplantation is not available. Peritoneal dialysis (PD) and hemodialysis (HD) are lifesaving RRTs for more than 2 million patients with ESRD worldwide [1]. As the incidence of chronic renal disease has doubled over the past decade, the number of patients with ESRD is expected to increase by 5–8% annually [2]. Although long-term morbidity and mortality are comparable between PD and HD, there is an early patient survival advantage for PD and a better quality of life [3]. Moreover PD is cost-effective as compared to hospital-based HD [4]. Nevertheless only one out of 10 patients is treated with PD, which suggests a general underutilization [5]. PD is a simple therapy in which PD fluid is exchanged several times a day; the major limitations are peritoneal membrane damage on the long-term and infection [6]. PD could be largely enhanced if one can identify diagnostic and therapeutic tools to improve PD outcome that promote function of the peritoneal membrane and prevent infectious complications [7]. European Training and Research in Peritoneal Dialysis (EuTRiPD) is an EU funded training programme, in which exactly these goals are targeted [8].

In this special issue of BMRI, we present a number of articles prepared in part (but also by other contributors) with a support from the EuTRiPD initiative. These are both original research papers and review papers that (i) analyze basic aspects of peritoneal cell biology using in vitro methods, (ii) mimic clinical situations in relevant PD models in rodents, and (iii) describe the outcomes of clinical studies using new biomarkers or new interventions.

In the following papers, firstly studies apply in vitro models to investigate the senescence-associated proteome in mesothelial cells, the effect of differently sterilized PD fluids on mesothelial stress responses, and the contribution of mesothelial-to-mesenchymal transition to peritoneal fibroblast expansion. Secondly with respect to papers in rodent PD models a new mouse uremic model is presented, the effect of rapamycin in a mouse model is shown, and moreover clear indications are described in protective effect of paricalcitol in a PD model and another PD-fibrosis-like model. Thirdly clinical material was used in studies showing the importance of, respectively, HA, microRNA, osmolarity of fluid, and tenascin-C levels reflecting peritoneal patient status. Finally underutilization of PD is discussed as originating from less education and referral pattern and moreover it is discussed that new biomarkers in PD could contribute significantly to making choices for the future as shown by EuTRiPD. The editors feel that all these papers give a good overview of the state of art in addressing all aspects of PD.

*Robert H. J. Beelen* *Donald J. Fraser* *Peter Rutherford* *Janusz Witowski*
